# (*E*)-*N*′-(4-Hy­droxy­benzyl­idene)-3-nitro­benzohydrazide

**DOI:** 10.1107/S1600536811051233

**Published:** 2011-12-03

**Authors:** Xu-Feng Meng, Dong-Yue Wang, Jing-Jun Ma

**Affiliations:** a Hebei Key Laboratory of Bioinorganic Chemistry, College of Sciences, Agricultural University of Hebei, Baoding 071001, People’s Republic of China

## Abstract

The mol­ecule of the title compound, C_14_H_11_N_3_O_4_, assumes an *E* conformation about the C=N double bond. The benzene rings form a dihedral angle of 3.9 (2)°. The crystal structure is stabilized by N—H⋯O, O—H⋯N, O—H⋯O and C—H⋯O hydrogen bonds, forming layers parallel to (101). In addition, intra­layer π–π stacking inter­actions [centroid–centroid distance = 3.635 (2) Å] are observed.

## Related literature

For the biological activity of benzohydrazide compounds, see: El-Sayed *et al.* (2011 ▶); Horiuchi *et al.* (2009 ▶). For coordination compounds of benzohydrazide derivatives, see: El-Dissouky *et al.* (2010 ▶); Zhang *et al.* (2010 ▶). For standard bond lengths, see: Allen *et al.* (1987 ▶). For similar structures, see: Liu *et al.* (2011 ▶); Zhou *et al.* (2011 ▶); Meng *et al.* (2011 ▶).
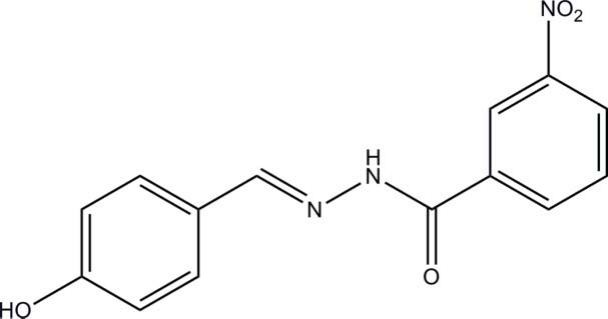


## Experimental

### 

#### Crystal data

C_14_H_11_N_3_O_4_
*M* *_r_* = 285.26
Monoclinic, 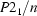

*a* = 10.362 (2) Å
*b* = 12.178 (3) Å
*c* = 10.468 (2) Å
β = 91.666 (2)°
*V* = 1320.3 (5) Å^3^
*Z* = 4
Mo *K*α radiation
μ = 0.11 mm^−1^
*T* = 298 K
0.17 × 0.15 × 0.15 mm

#### Data collection

Bruker SMART 1K CCD area-detector diffractometer
Absorption correction: multi-scan (*SADABS*; Sheldrick, 1996 ▶) *T*_min_ = 0.982, *T*_max_ = 0.984
10464 measured reflections
2884 independent reflections
2017 reflections with *I* > 2σ(*I*)
*R*_int_ = 0.034

#### Refinement

*R*[*F* ^2^ > 2σ(*F* ^2^)] = 0.043
*wR*(*F* ^2^) = 0.129
*S* = 1.03
2884 reflections
194 parameters
1 restraint
H atoms treated by a mixture of independent and constrained refinement
Δρ_max_ = 0.20 e Å^−3^
Δρ_min_ = −0.23 e Å^−3^

### 

Data collection: *SMART* (Bruker, 2007 ▶); cell refinement: *SAINT* (Bruker, 2007 ▶); data reduction: *SAINT*; program(s) used to solve structure: *SHELXS97* (Sheldrick, 2008 ▶); program(s) used to refine structure: *SHELXL97* (Sheldrick, 2008 ▶); molecular graphics: *SHELXTL* (Sheldrick, 2008 ▶); software used to prepare material for publication: *SHELXTL*.

## Supplementary Material

Crystal structure: contains datablock(s) I, global. DOI: 10.1107/S1600536811051233/rz2676sup1.cif

Structure factors: contains datablock(s) I. DOI: 10.1107/S1600536811051233/rz2676Isup2.hkl

Supplementary material file. DOI: 10.1107/S1600536811051233/rz2676Isup3.cml

Additional supplementary materials:  crystallographic information; 3D view; checkCIF report

## Figures and Tables

**Table 1:** Hydrogen-bond geometry (Å, °)

*D*—H⋯*A*	*D*—H	H⋯*A*	*D*⋯*A*	*D*—H⋯*A*
O1—H1⋯O2^i^	0.82	2.02	2.8341 (18)	170
O1—H1⋯N1^i^	0.82	2.58	3.0757 (19)	120
N2—H2*A*⋯O1^ii^	0.89 (1)	2.53 (2)	3.0597 (19)	119 (2)
C5—H5⋯O1^iii^	0.93	2.54	3.367 (2)	147

Symmetry codes: (i) 
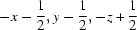
; (ii) 
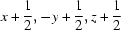
; (iii) 
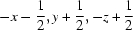
.

**Fig. 1.**
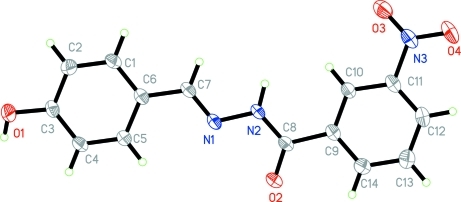
The molecular structure of the title compound with displacement ellipsoids drawn at the 30% probability level.

**Fig. 2.**
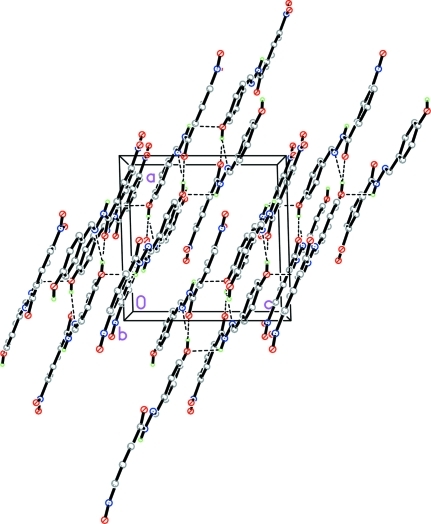
The crystal packing of the title compound, showing the N—H···O, O—H···N, and O—H···O hydrogen-bonds (dashed lines). H-atoms not involved in the hydrogen bonding have been omitted for clarity.

## supplementary crystallographic information

### Comment

Benzohydrazide compounds are well known for their biological activities (El-Sayed *et al.*, 2011; Horiuchi *et al.*, 2009). In addition, benzohydrazide compounds have also been used as versatile ligands in coordination chemistry (El-Dissouky *et al.*, 2010, Zhang *et al.*, 2010). As a contribution to the structural study of hydrazone compounds, we present here the crystal structure of the title compound, which was obtained as the product of the reaction of 4-hydroxybenzaldehyde with 3-nitrobenzohydrazide in methanol.

In the title compound, Fig. 1, the mean planes of the two benzene rings form a dihedral angle of 3.9 (2)°. The bond distances and angles are within normal ranges (Allen *et al.*, 1987), and agree well with the corresponding bond distances and angles reported in closely related compounds (Meng *et al.*, 2011; Liu *et al.*, 2011; Zhou *et al.*, 2011). In the crystal structure, intermolecular N—H···O, O—H···N, C—H···O and O—H···O hydrogen bonds (Table 1; Fig. 2) link molecules into layers parallel to the (101) plane. The layers are further stabilized by π–π stacking interactions with centroid-to-centroid distances of 3.635 (2) Å.

### Experimental

To a methanol solution (20 ml) of 4-hydroxybenzaldehyde (0.1 mmol, 12.2 mg) and 3-nitrobenzohydrazide (0.1 mmol, 18.1 mg), a few drops of acetic acid were added. The mixture was refluxed for 1 h and then cooled to room temperature. The yellow crystalline solid was collected by filtration, washed with cold methanol and dried in air. Single crystals, suitable for X-ray diffraction, were obtained by slow evaporation of a methanol solution of the product in air.

### Refinement

The imine H atoms was located in a difference Fourier map and refined with the N—H distance restrained to 0.90 (1) Å and with *U*_iso_(H) = 0.08 Å^2^. The C- and O-bound H atoms were positioned geometrically and refined using a riding model, with C—H = 0.93 Å, O—H = 0.82 Å, and with *U*_iso_(H) = 1.2 *U*_eq_(C) or 1.5 *U*_eq_(O).

### Crystal data

**Table:**

C_14_H_11_N_3_O_4_	*F*(000) = 592
*M**_r_* = 285.26	*D*_x_ = 1.435 Mg m^−^^3^
Monoclinic, *P*2_1_/*n*	Mo *K*α radiation, λ = 0.71073 Å
Hall symbol: -P 2yn	Cell parameters from 3224 reflections
*a* = 10.362 (2) Å	θ = 2.5–27.2°
*b* = 12.178 (3) Å	µ = 0.11 mm^−^^1^
*c* = 10.468 (2) Å	*T* = 298 K
β = 91.666 (2)°	Block, yellow
*V* = 1320.3 (5) Å^3^	0.17 × 0.15 × 0.15 mm
*Z* = 4	

### Data collection

**Table:**

Bruker SMART 1K CCD area-detector diffractometer	2884 independent reflections
Radiation source: fine-focus sealed tube	2017 reflections with *I* > 2σ(*I*)
graphite	*R*_int_ = 0.034
ω scan	θ_max_ = 27.0°, θ_min_ = 2.6°
Absorption correction: multi-scan (*SADABS*; Sheldrick, 1996)	*h* = −11→13
*T*_min_ = 0.982, *T*_max_ = 0.984	*k* = −15→15
10464 measured reflections	*l* = −13→13

### Refinement

**Table:**

Refinement on *F*^2^	Primary atom site location: structure-invariant direct methods
Least-squares matrix: full	Secondary atom site location: difference Fourier map
*R*[*F*^2^ > 2σ(*F*^2^)] = 0.043	Hydrogen site location: inferred from neighbouring sites
*wR*(*F*^2^) = 0.129	H atoms treated by a mixture of independent and constrained refinement
*S* = 1.03	*w* = 1/[σ^2^(*F*_o_^2^) + (0.0581*P*)^2^ + 0.233*P*] where *P* = (*F*_o_^2^ + 2*F*_c_^2^)/3
2884 reflections	(Δ/σ)_max_ < 0.001
194 parameters	Δρ_max_ = 0.20 e Å^−^^3^
1 restraint	Δρ_min_ = −0.23 e Å^−^^3^

### Special details

**Table:**

Geometry. All e.s.d.'s (except the e.s.d. in the dihedral angle between two l.s. planes) are estimated using the full covariance matrix. The cell e.s.d.'s are taken into account individually in the estimation of e.s.d.'s in distances, angles and torsion angles; correlations between e.s.d.'s in cell parameters are only used when they are defined by crystal symmetry. An approximate (isotropic) treatment of cell e.s.d.'s is used for estimating e.s.d.'s involving l.s. planes.
Refinement. Refinement of *F*^2^ against ALL reflections. The weighted *R*-factor *wR* and goodness of fit *S* are based on *F*^2^, conventional *R*-factors *R* are based on *F*, with *F* set to zero for negative *F*^2^. The threshold expression of *F*^2^ > σ(*F*^2^) is used only for calculating *R*-factors(gt) *etc*. and is not relevant to the choice of reflections for refinement. *R*-factors based on *F*^2^ are statistically about twice as large as those based on *F*, and *R*- factors based on ALL data will be even larger.

### Fractional atomic coordinates and isotropic or equivalent isotropic displacement parameters (Å^2^)

**Table:**

	*x*	*y*	*z*	*U*_iso_*/*U*_eq_	
N1	0.05181 (12)	0.48482 (12)	0.34202 (13)	0.0426 (4)	
N2	0.14827 (13)	0.55140 (12)	0.39294 (14)	0.0432 (4)	
N3	0.56139 (14)	0.71802 (15)	0.60566 (14)	0.0521 (4)	
O1	−0.27851 (12)	0.07966 (10)	0.13367 (13)	0.0548 (4)	
H1	−0.3510	0.1064	0.1273	0.082*	
O2	0.01462 (11)	0.69680 (10)	0.38555 (12)	0.0525 (4)	
O3	0.57302 (14)	0.61913 (14)	0.62040 (16)	0.0768 (5)	
O4	0.64912 (12)	0.78320 (13)	0.62796 (14)	0.0682 (4)	
C1	0.01449 (16)	0.19488 (15)	0.27428 (16)	0.0451 (4)	
H1A	0.0946	0.1703	0.3047	0.054*	
C2	−0.07416 (16)	0.12048 (15)	0.22597 (17)	0.0481 (4)	
H2	−0.0539	0.0461	0.2240	0.058*	
C3	−0.19415 (15)	0.15658 (14)	0.18014 (15)	0.0403 (4)	
C4	−0.22330 (16)	0.26705 (14)	0.18248 (16)	0.0418 (4)	
H4	−0.3030	0.2916	0.1510	0.050*	
C5	−0.13454 (15)	0.34089 (14)	0.23140 (15)	0.0414 (4)	
H5	−0.1552	0.4152	0.2333	0.050*	
C6	−0.01402 (15)	0.30600 (14)	0.27822 (14)	0.0383 (4)	
C7	0.07926 (15)	0.38382 (14)	0.33207 (15)	0.0416 (4)	
H7	0.1603	0.3590	0.3596	0.050*	
C8	0.12135 (15)	0.65778 (14)	0.41169 (14)	0.0387 (4)	
C9	0.22883 (15)	0.72875 (14)	0.46301 (14)	0.0387 (4)	
C10	0.34487 (15)	0.68786 (14)	0.51232 (14)	0.0403 (4)	
H10	0.3601	0.6126	0.5155	0.048*	
C11	0.43746 (15)	0.76159 (15)	0.55668 (15)	0.0425 (4)	
C12	0.41881 (18)	0.87291 (16)	0.55569 (17)	0.0524 (5)	
H12	0.4824	0.9202	0.5875	0.063*	
C13	0.30287 (19)	0.91322 (16)	0.5062 (2)	0.0599 (5)	
H13	0.2880	0.9885	0.5038	0.072*	
C14	0.20934 (17)	0.84119 (15)	0.46041 (17)	0.0499 (4)	
H14	0.1317	0.8689	0.4272	0.060*	
H2A	0.2202 (14)	0.5183 (17)	0.4207 (19)	0.080*	

### Atomic displacement parameters (Å^2^)

**Table:**

	*U*^11^	*U*^22^	*U*^33^	*U*^12^	*U*^13^	*U*^23^
N1	0.0309 (7)	0.0467 (9)	0.0495 (8)	−0.0055 (6)	−0.0112 (6)	−0.0001 (6)
N2	0.0285 (7)	0.0449 (8)	0.0553 (8)	−0.0034 (6)	−0.0141 (6)	0.0009 (6)
N3	0.0346 (8)	0.0727 (12)	0.0487 (8)	−0.0096 (8)	−0.0060 (6)	0.0011 (8)
O1	0.0401 (7)	0.0508 (8)	0.0729 (8)	−0.0085 (6)	−0.0092 (6)	−0.0107 (6)
O2	0.0333 (7)	0.0542 (8)	0.0691 (8)	0.0028 (5)	−0.0139 (6)	−0.0007 (6)
O3	0.0500 (9)	0.0740 (11)	0.1050 (12)	−0.0048 (8)	−0.0232 (8)	0.0193 (9)
O4	0.0378 (7)	0.0908 (11)	0.0752 (9)	−0.0179 (7)	−0.0097 (6)	−0.0121 (8)
C1	0.0300 (8)	0.0517 (10)	0.0530 (10)	0.0035 (7)	−0.0052 (7)	−0.0029 (8)
C2	0.0411 (10)	0.0420 (10)	0.0612 (11)	0.0019 (8)	−0.0009 (8)	−0.0050 (8)
C3	0.0320 (8)	0.0477 (10)	0.0411 (8)	−0.0081 (7)	−0.0005 (6)	−0.0053 (7)
C4	0.0308 (8)	0.0495 (10)	0.0448 (9)	−0.0003 (7)	−0.0068 (7)	0.0010 (7)
C5	0.0351 (9)	0.0411 (9)	0.0475 (9)	−0.0014 (7)	−0.0055 (7)	−0.0011 (7)
C6	0.0304 (8)	0.0469 (10)	0.0373 (8)	−0.0033 (7)	−0.0009 (6)	−0.0028 (7)
C7	0.0279 (8)	0.0506 (11)	0.0460 (9)	−0.0017 (7)	−0.0060 (7)	−0.0011 (7)
C8	0.0307 (8)	0.0483 (10)	0.0368 (8)	−0.0015 (7)	−0.0055 (6)	0.0035 (7)
C9	0.0322 (8)	0.0474 (10)	0.0364 (8)	−0.0046 (7)	−0.0009 (6)	0.0006 (7)
C10	0.0325 (9)	0.0468 (10)	0.0415 (8)	−0.0050 (7)	−0.0038 (7)	0.0014 (7)
C11	0.0313 (9)	0.0594 (11)	0.0366 (8)	−0.0079 (8)	−0.0011 (6)	0.0007 (7)
C12	0.0457 (10)	0.0558 (12)	0.0554 (11)	−0.0154 (9)	−0.0028 (8)	−0.0068 (9)
C13	0.0567 (12)	0.0468 (11)	0.0757 (13)	−0.0052 (9)	−0.0052 (10)	−0.0059 (9)
C14	0.0432 (10)	0.0497 (11)	0.0563 (10)	0.0030 (8)	−0.0057 (8)	−0.0015 (8)

### Geometric parameters (Å, °)

**Table:**

N1—C7	1.267 (2)	C4—H4	0.9300
N1—N2	1.3819 (18)	C5—C6	1.394 (2)
N2—C8	1.341 (2)	C5—H5	0.9300
N2—H2A	0.888 (9)	C6—C7	1.456 (2)
N3—O3	1.220 (2)	C7—H7	0.9300
N3—O4	1.224 (2)	C8—C9	1.497 (2)
N3—C11	1.468 (2)	C9—C14	1.384 (2)
O1—C3	1.3614 (19)	C9—C10	1.387 (2)
O1—H1	0.8200	C10—C11	1.384 (2)
O2—C8	1.2272 (18)	C10—H10	0.9300
C1—C2	1.376 (2)	C11—C12	1.369 (3)
C1—C6	1.386 (2)	C12—C13	1.384 (3)
C1—H1A	0.9300	C12—H12	0.9300
C2—C3	1.391 (2)	C13—C14	1.383 (2)
C2—H2	0.9300	C13—H13	0.9300
C3—C4	1.379 (2)	C14—H14	0.9300
C4—C5	1.375 (2)		
			
C7—N1—N2	116.07 (13)	C5—C6—C7	121.03 (15)
C8—N2—N1	118.17 (13)	N1—C7—C6	121.04 (15)
C8—N2—H2A	124.5 (15)	N1—C7—H7	119.5
N1—N2—H2A	116.8 (15)	C6—C7—H7	119.5
O3—N3—O4	123.09 (17)	O2—C8—N2	122.10 (15)
O3—N3—C11	118.90 (15)	O2—C8—C9	120.85 (16)
O4—N3—C11	118.00 (17)	N2—C8—C9	117.03 (14)
C3—O1—H1	109.5	C14—C9—C10	119.13 (15)
C2—C1—C6	120.87 (15)	C14—C9—C8	117.22 (15)
C2—C1—H1A	119.6	C10—C9—C8	123.65 (15)
C6—C1—H1A	119.6	C11—C10—C9	118.48 (16)
C1—C2—C3	119.95 (16)	C11—C10—H10	120.8
C1—C2—H2	120.0	C9—C10—H10	120.8
C3—C2—H2	120.0	C12—C11—C10	122.91 (16)
O1—C3—C4	122.60 (15)	C12—C11—N3	118.83 (15)
O1—C3—C2	117.65 (16)	C10—C11—N3	118.26 (16)
C4—C3—C2	119.75 (15)	C11—C12—C13	118.34 (16)
C5—C4—C3	120.01 (15)	C11—C12—H12	120.8
C5—C4—H4	120.0	C13—C12—H12	120.8
C3—C4—H4	120.0	C14—C13—C12	119.78 (18)
C4—C5—C6	120.95 (16)	C14—C13—H13	120.1
C4—C5—H5	119.5	C12—C13—H13	120.1
C6—C5—H5	119.5	C13—C14—C9	121.35 (17)
C1—C6—C5	118.46 (14)	C13—C14—H14	119.3
C1—C6—C7	120.50 (14)	C9—C14—H14	119.3

### Hydrogen-bond geometry (Å, °)

**Table:**

*D*—H···*A*	*D*—H	H···*A*	*D*···*A*	*D*—H···*A*
O1—H1···O2^i^	0.82	2.02	2.8341 (18)	170.
O1—H1···N1^i^	0.82	2.58	3.0757 (19)	120.
N2—H2A···O1^ii^	0.89 (1)	2.53 (2)	3.0597 (19)	119.(2)
C5—H5···O1^iii^	0.93	2.54	3.367 (2)	147

Symmetry codes: (i) −*x*−1/2, *y*−1/2, −*z*+1/2; (ii) *x*+1/2, −*y*+1/2, *z*+1/2; (iii) −*x*−1/2, *y*+1/2, −*z*+1/2.

## References

[bb1] Allen, F. H., Kennard, O., Watson, D. G., Brammer, L., Orphen, A. G. & Taylor, R. (1987). *J. Chem. Soc. Perkin Trans. 2*, pp. S1–19.

[bb2] Bruker (2007). *SMART* and *SAINT* Bruker AXS Inc., Madison, Wisconsin, USA.

[bb3] El-Dissouky, A., Al-Fulaij, O., Awad, M. K. & Rizk, S. (2010). *J. Coord. Chem.* **63**, 330–345.

[bb4] El-Sayed, M. A. A., Abdel-Aziz, N. I., Abdel-Aziz, A. A. M., El-Azab, A. S., Asiri, Y. A. & ElTahir, K. E. H. (2011). *Bioorg. Med. Chem.* **19**, 3416–3424. 10.1016/j.bmc.2011.04.027 21570309

[bb5] Horiuchi, T., Nagata, M., Kitagawa, M., Akahane, K. & Uoto, K. (2009). *Bioorg. Med. Chem.* **17**, 7850–7860. 10.1016/j.bmc.2009.10.039 19889545

[bb6] Liu, W.-H., Song, S.-J. & Ma, J.-J. (2011). *Acta Cryst.* E**67**, o2198. 10.1107/S1600536811030108 PMC3213628 22091205

[bb7] Meng, X.-F., Wang, D.-Y. & Ma, J.-J. (2011). *Acta Cryst.* E**67**, o3109. 10.1107/S1600536811044291 PMC3247494 22220112

[bb8] Sheldrick, G. M. (1996). *SADABS* University of Göttingen, Germany.

[bb9] Sheldrick, G. M. (2008). *Acta Cryst.* A**64**, 112–122. 10.1107/S0108767307043930 18156677

[bb10] Zhang, S.-P., Wei, Y. & Shao, S.-C. (2010). *Acta Cryst.* E**66**, m1635. 10.1107/S1600536810047719 PMC3011487 21589308

[bb11] Zhou, X., Gao, S.-T. & Ma, J.-J. (2011). *Acta Cryst.* E**67**, o2275. 10.1107/S1600536811031187 PMC3200651 22065140

